# Our Contemporaries

**Published:** 1916-06

**Authors:** 


### OUR CONTEMPORARIES

---

The Illinois Medical Journal, May, comments editorially on the proposed national licensing board. It agrees with our contention that such a board cannot be established without a constitutional amendment, which would take this power from the states and with it, either by general wording or by precedent, other powers. It discusses a proposition to establish a voluntary licensing board which we confess, we do not comprehend. We do not see how a national licensing board can be in any way unofficial. Of course, any state board could voluntarily submit to the findings of such a board but so they can extend their reciprocity. The Illinois

Medical Journal concedes the broad advantages of a national license and believes that some way can be found to solve the problem by co-operation of states. With all this, we heartily agree, though we admit a personal belief which we do not consider proper as a journalistic policy, that it is high time that this country had a single government, except for purely local matters for small areas such as counties and cities. This belief may not be so nefarious as it seems at first thought. At any rate, many of the framers of the Constitution regarded the maintenance of the states as a temporary expedient.

